# Putative role of protein kinase C in neurotoxic inflammation mediated by extracellular heat shock protein 70 after ischemia-reperfusion

**DOI:** 10.1186/1742-2094-11-81

**Published:** 2014-04-23

**Authors:** Galina Dvoriantchikova, Andrea Rachelle C Santos, Ali M Saeed, Xenia Dvoriantchikova, Dmitry Ivanov

**Affiliations:** 1 Bascom Palmer Eye Institute, Department of Ophthalmology, University of Miami Miller School of Medicine, 1638 NW 10th Ave, Miami, FL 33136, USA; 2 Sheila and David Fuente Program in Cancer Biology, University of Miami Miller School of Medicine, 1550 NW 10th Ave, Miami, FL 33136, USA; 3 Department of Microbiology and Immunology, University of Miami Miller School of Medicine, 1600 NW 10th Ave, Miami, FL 33136, USA

**Keywords:** Ischemia-reperfusion (IR) injury, Sterile inflammation, Damage-associated molecular patterns (DAMPs), Extracellular heat shock protein 70 (Hsp70), Toll-like receptor 4 (Tlr4), Endotoxins, Polymyxin B, Protein kinase C (PKC), Neuronal death

## Abstract

**Background:**

Sterile inflammation occurs in the absence of live pathogens and is an unavoidable consequence of ischemia-reperfusion (IR) injury in the central nervous system (CNS). It is known that toll-like receptor 4 (Tlr4) contributes to damage and sterile inflammation in the CNS mediated by IR. However, the mechanism of Tlr4 activation under sterile conditions in ischemic tissue is poorly understood. We performed this study to clarify the mechanism. To this end, we focused on the extracellular heat shock protein 70 (Hsp70), the prototypic Tlr4 ligand.

**Methods:**

Tlr4-, Myd88- and Trif-knockout animals, as well as C57BL/6 mice, were used for the wild type control. For the *in vivo* study, we used a mouse model of retinal IR injury. To test the role of protein kinase C (PKC) in IR injury, IR retinas were treated with the PKC inhibitors (polymyxin B and Gö6976) and retinal damage was evaluated by directly counting neurons in the ganglion cell layer of flat-mounted retinas seven days after IR. Primary retinal neurons (retinal ganglion cells) and glial cells were used for *in vitro* experiments. Quantitative RT-PCR, ELISA and western blot analysis were used to study the production of pro-inflammatory factors in IR retinas and in primary cell cultures.

**Results:**

We found significant accumulation of extracellular Hsp70 in a model of retinal IR injury. We noted that PKC was involved in Tlr4 signaling, and found that PKC inhibitors promoted neuroprotection by reducing pro-inflammatory activity in ischemic tissue. To put all of the pieces in the signaling cascade together, we performed an *in vitro* study. We found that PKC was critical to mediate the Hsp70-dependent pro-inflammatory response. At the same time, the contamination of Hsp70 preparations with low-dose endotoxin was not critical to mediate the production of pro-inflammatory factors. We found that extracellular Hsp70 can promote neuronal death at least, by mediating production of cytotoxic levels of tumor necrosis factor alpha, predominantly due to the Tlr4/Myd88 signaling cascade.

**Conclusions:**

Our findings suggest that PKC acts as a switch to amplify the pro-inflammatory activity of Hsp70/Tlr4 signaling, which is sufficient to mediate neuronal death.

## Background

Sterile inflammation, characterized by innate immune responses in tissue, occurs in the absence of live pathogens, is a pathologic hallmark of ischemia-reperfusion (IR) injury in the central nervous system (CNS), and is spatiotemporally related to delayed neuronal death [1-3]. It has been reported that endogenous ligands (damage-associated molecular patterns (DAMPs)) liberated from IR injured cells act through pattern recognition receptors, initiating an innate immune response in tissue [4-9]. Toll-like receptor 4 (Tlr4) is a pattern recognition receptor that initiates an innate immune response in the presence of exogenous (bacteria-derived endotoxins) and endogenous (DAMPs) ligands [9,10]. Tlr4 plays a critical role in mediating CNS sterile inflammation and tissue damage triggered by IR [11,12]. However, the mechanism by which DAMPs activate Tlr4 was not understood. This issue creates evident challenges for the development of drugs that are effective against IR injury.

Currently, there are conflicting results regarding whether or not DAMPs can directly activate Tlr4. While it was shown that DAMPs or their modifications can directly activate Tlr4 [3,9,13,14], there is evidence that DAMPs can initiate an innate immune response acting in synergy with low-dose endotoxin [15-17]. Since the presence of low endotoxin levels has been reported in many tissues, including the CNS [18-21], it could be possible that DAMPs released after IR combine with endotoxins and cooperate to initiate a pro-inflammatory response that promotes neuronal death and tissue damage. At the same time, many of the studies that argue that DAMPs work with endotoxins use an endotoxin inhibitor named Polymyxin B (PMB) [22]. It is important to note that PMB is also a protein kinase C (PKC) inhibitor [23-25]. It is known that PKC acts at multiple steps of toll-like receptor signaling, promoting an innate immune response in tissue [26]. Thus, DAMP-dependent pro-inflammatory toxicity in ischemic tissue could be due to PKC activity. In this study, we explore whether DAMPs released after IR promote neuronal toxicity alone, because of endotoxin presence, or as a result of PKC activity. To figure out an answer, we focused on the extracellular heat shock protein 70 (Hsp70), the prototypic DAMP molecule and ligand of Tlr4 [3,9,14]. Our findings suggest that Hsp70 released after IR can initiate innate immune-mediated neurotoxicity in a PKC-dependent manner. These findings also propose that the presence of low endotoxin levels in Hsp70 preparations is not critical in mediating the production of pro-inflammatory factors.

## Methods

### Materials

All chemicals and reagents were purchased from Sigma-Aldrich (St. Louis, Missouri, United States, Life Technologies (Grand Island, New York, United States), Thermo Scientific (Rockford, Illinois, United States) and Lonza (Walkersville, Maryland, United States). Polymyxin B sulfate was purchased from Santa Cruz Biotechnology (Dallas, Texas, United States), recombinant low endotoxin HSP70 (Cat# ADI-ESP-555-F, < 50 EU/mg purified protein) was purchased from Enzo Life Sciences (Farmingdale, New York, United States). RAW-BlueTM cells (RAW264.7 macrophages stably transfected with the NF-κB reporter gene) were purchased from InvivoGen (San Diego, California, United States). Lipopolysaccharides (LPS) from Escherichia coli 0111:B4 were purchased from Sigma-Aldrich (St. Louis, Missouri, United States).

### Animals

All experiments and postsurgical care were performed in compliance with the National Institutes of Health (NIH) Guide for the Care and Use of Laboratory Animals and according to the University of Miami Institutional Animal Care and Use Committee (IACUC) approved protocols. Tlr4-, Myd88-, and Trif-deficient animals (stock numbers 007227; 009088, 005037) and C57BL/6 J (stock number 000664) mice as the wild type (WT) control were obtained from the Jackson Laboratory (Bar Harbor, Maine, United States). Mice were housed under standard conditions of temperature and humidity, with a 12 hour light to dark cycle and free access to food and water. All animals used in our experiments were either 3-month old male mice, or 3 or 12-day old pups.

### Transient retinal ischemia

Anesthesia was induced with isoflurane and maintained for 45 minutes. Anesthetics were administered to the breathing animals through a tightly fitting nose cone. Body temperature was held constant at 37°C with a temperature-controlled heating pad. After anesthesia, pupils were dilated with 1% tropicamide to 2.5% phenylephrine hydrochloride (NutraMax Products Inc., Gloucester, Massachusetts, United States), and corneal analgesia was achieved with one drop of 0.5% proparacaine HCl (Bausch and Lomb Pharmaceuticals, Tampa, Florida, United States). Retinal ischemia was induced for 45 minutes by introducing into the anterior chamber of the left eye a 33-gauge needle attached to a normal (0.9% NaCl) saline-filled reservoir raised above the animal in order to increase intraocular pressure (IOP, increased to 120 mmHg). The contralateral eye was cannulated and maintained at normal IOP to serve as a normotensive control. Complete retinal ischemia, evidenced by a whitening of the anterior segment of the eye and blanching of the retinal arteries, was verified by microscopic examination. After the needle was removed, erythromycin ophthalmic ointment (Fera Pharmaceuticals, Locust Valley, New York, United States) was applied to the conjunctival sac. Mice were euthanized by CO_2_ inhalation under anesthesia.

### Treatment of animals with Polymixin B and Gö6976

Mice were injected intraperitoneally (IP) with either PMB (8 μg/g body of mouse) or Gö6976 (167 ng/g body of mouse it is fine to leave as is) one hour before ischemia, six hours after surgery, and twice every twenty four hours until they were euthanized. Control animals were treated with phosphate-buffered saline (PBS). To evaluate the real concentration of PMB and Gö6976 in the retina, we performed the following calculations. After the IP injection, the drug was distributed throughout the animal’s body and accumulated in the tissues and organs. Thus, we had to take into account the volume of the whole animal body in our calculations. The average volume of the adult mice used in our study was 30 ml (we used Archimedes’ principle to measure this volume, by immersing the body of the mouse in water and measuring volume of water displacement, which equals volume of the mouse’s body)). To calculate the PMB concentration, we took into account: 1) mouse volume = 30 ml; 2) PMB molecular weight = 1301.56 g/mol; 3) we injected 8 μg of PMB per 1 g of mouse; 4) average mouse weight = 30 g. Thus, we injected a total of 240 μg of PMB per one injection (30 (g) × 8 (μg of PMB per 1 g of mouse) = 240 μg). We injected 240 × 10^−6^ (g of PMB)/1301.56 (g/mol) = 1.84 × 10^−7^ mol. Therefore, the concentration was 1.84 × 10^−7^ (mol)/(30 × 10^−3^ L) = 6.1 × 10^−6^ (mol/L) = 6 μM. However, we took into account that the total water content of the mouse was about 80%, and so the final concentration was 7.5 μM. By performing similar calculations and considering that Gö6976’s molecular weight was 378.43 g/mol, we calculated the final concentration of Gö6976, which was equal to 551 nM. Importantly, since mice were injected intraperitoneally (IP) twice every twenty four hours until they were euthanized seven days later (after reperfusion), the real concentration of PMB and Gö6976 in the retina could be higher.

### Immunohistochemistry

Eyes were enucleated upon euthanasia, incised at the ora serrate, and immersion-fixed in 4% PF. After one hour, the retinas were removed and cryoprotected overnight in 30% sucrose. The following day, the retinas underwent three freeze–thaw cycles, were rinsed 3 × 10 minutes in 0.1 M Tris buffer, were blocked by 5% donkey serum and 0.1% Triton X-100 in 0.1 M Tris buffer for one hour, and were then incubated overnight with either monoclonal FITC–conjugated neuronal nuclei (NeuN) antibody (1:300; Chemicon, Billerica, Massachusetts, United States) or beta III Tubulin antibody (1:250; Covance, Denver, Pennsylvania, United States). After 3 × 10 minutes rinsing in 0.1 M Tris buffer, the retinas were flat-mounted, cover-slipped, and imaged using a Leica TSL AOBS SP5 confocal microscope (Leica Microsystems, Exton, Pennsylvania, United States).

### Counting of ganglion cell layer neurons

NeuN- or beta III Tubulin-positive neurons in the ganglion cell layer (GCL) were imaged by confocal microscopy in flat-mounted retinas. Individual retinas were sampled randomly to collect a total of 20 images from four retinal quadrants using a 20× objective lens. Five images were collected from each quadrant: one from the center, two from the middle, and two from the peripheral regions of the retina. NeuN-positive neurons were counted using ImageJ software. This software counts cells semi-automatically, meaning that it records the number of cells that are clicked on by the person identifying cells in the image. Cell loss in the ischemic retinas was calculated as a percentage of the mean cell density in normotensive fellow control eyes.

### NF-κB reporter assay

RAW-Blue cells (RAW264.7 macrophages stably expressed a secreted embryonic alkaline phosphatase gene inducible by NF-κB and resistant to the selectable marker Zeocin) were seeded in 96-well plates in test medium (DMEM, 4.5 g/l glucose, 10% heat-inactivated FBS, 100 mg/ml Normocin™, 2 mM L-glutamine) at a density of 4 × 10^5^ cells/ml and grown overnight in a 5% CO_2_ incubator at 37°C. After treated as indicated, the medium was harvested, and 20-μl samples were mixed with the QUANTI-Blue™ (InvivoGen, San Diego, California, United States) medium (200 μl) in 96-well plates at 37°C for 15 minutes, and the optical density at 655 nm was measured on a Multiskan microplate spectrophotometer (Thermo Scientific, Brookfield, Wisconsin, United States).

### Primary cultures of retinal ganglion cells, astrocytes and microglia

To isolate retinal ganglion cells (RGCs), we used the two-step immunopanning protocol [8,27]. To this end, the retinas were incubated in papain solution (16.5 U/mL) for 30 minutes. Next, macrophage and endothelial cells were removed from the cell suspension by panning with the anti-macrophage antiserum (Accurate Chemical, Westbury, New York, United States). RGCs were bound to the panning plates containing anti-Thy1.2 antibody and released by trypsin incubation. Isolated primary RGCs were grown in serum-free media (Neurobasal/B27 media; Life Technologies, Grand Island, New York, United States) one day before the experiment. Astrocytes and microglial cells were obtained by the ‘shaking method’ as previously described [28]. These cells were cultured in DMEM (Life Technologies, Grand Island, New York, United States) containing 10% heat-inactivated fetal bovine serum (FBS) (Life Technologies, Grand Island, New York, United States) one day before the experiment. Astrocytes and microglial cells were maintained during the experiment in DMEM containing 0.1% FBS. To prepare RGC/glia co-culture, RGCs were plated on cover slips in 24-well plate. Glia were plated in culture inserts and placed into the culture wells containing RGCs 24 hours prior to treatment. RGC/glia co-cultures containing 0.1% FBS were maintained during the experiment in DMEM.

### Neuronal death assay

After treatment, dead neurons were determined using a kit (Vybrant Apoptosis Assay Kit #2; Life Technologies, Grand Island, New York, United States). Cells were imaged using a confocal microscope (Leica TSL AOBS SP5; Leica Microsystems, Pennsylvania, United States). Individual glasses were sampled randomly to collect a total of 10 images using a 20X objective lens. The dead RGCs were counted using ImageJ software. The percentage of dead RGCs relative to the total number of cells was determined. The experiment was repeated at least three times.

### Quantitative reverse transcription polymerase chain reaction (RT-PCR) analysis

To perform quantitative RT-PCR analysis using gene-specific primers (Table 1), total RNA was extracted from retinas and primary cell cultures using Absolutely RNA Nanoprep or Microprep kits (Agilent Technologies, Santa Clara, California, United States) and reverse transcribed with the Reverse Transcription System (Promega, Madison, Wisconsin, United States) to synthesize cDNA. Quantitative PCR was performed in the Rotor-Gene Q Cycler (Qiagen, Valencia, California, United States) using the SYBR GREEN PCR MasterMix (Qiagen, Valencia, California, United States). For each gene, relative expression was calculated by comparison with a standard curve, following normalization to the housekeeping gene β-actin (*Actb*) and succinate dehydrogenase subunit A (*Sdha*) expression chosen as controls.

**Table 1:** List of PCR primers

**Gene**	**Oligonucleotides**	
***Myd88***	Forward	CTTGATGACCCCCTAGGACA
	Reverse	AGGCTGAGTGCAAACTTGGT
***Trif***	Forward	AGACCCCTACAGCCAGGTCT
	Reverse	GGCATGGAGAAGCTTTGACT
***Ripk1***	Forward	TGATGCACGTGCTAAAGACC
	Reverse	TGTTCGGGTGCCATGTAGTA
***Il1b***	Forward	GACCTTCCAGGATGAGGACA
	Reverse	AGGCCACAGGTATTTTGTCG
***Tnf***	Forward	CAAAATTCGAGTGACAAGCCTG
	Reverse	GAGATCCATGCCGTTGGC
***Ccl2***	Forward	AGGTCCCTGTCATGCTTCTG
	Reverse	ATTTGGTTCCGATCCAGGTT
***Ccl5***	Forward	AGCAGCAAGTGCTCCAATCT
	Reverse	ATTTCTTGGGTTTGCTGTGC
***Cxcl10***	Forward	GCTGCAACTGCATCCATATC
	Reverse	CACTGGGTAAAGGGGAGTGA
***Icam1***	Forward	TGGTGATGCTCAGGTATCCA
	Reverse	CACACTCTCCGGAAACGAAT
***Ncf1***	Forward	CGAGAAGAGTTCGGGAACAG
	Reverse	AGCCATCCAGGAGCTTATGA
***Nox2***	Forward	GACTGCGGAGAGTTTGGAAG
	Reverse	ACTGTCCCACCTCCATCTTG
***Nos2***	Forward	CAGAGGACCCAGAGACAAGC
	Reverse	TGCTGAAACATTTCCTGTGC
***Sdha***	Forward	ACACAGACCTGGTGGAGACC
	Reverse	GCACAGTCAGCCTCATTCAA
***Actb***	Forward	CACCCTGTGCTGCTCACC
	Reverse	GCACGATTTCCCTCTCAG

### Western blot analysis

Astrocyte monolayers were lysed with RIPA **(**named after its original application, radio-immunoprecipitation assay) buffer supplemented with complete protease inhibitor (Roche Applied Science, Indianapolis, Indiana, United States). Protein concentration was assessed using the BCA kit (Thermo Scientific, Brookfield, Wisconsin, United States). An equal amount of total protein from each sample was resolved on SDS-PAGE gradient gels and transferred to PVDF (polyvinylidene fluoride) membrane (Life Technologies, Grand Island, New York, United States). Blots were blocked in 5% milk in Tris-buffered saline (TBS, pH 7.6), probed with the primary antibody against Il1b (1:1,000, Abcam, Cambridge, Massachusetts, United States) overnight, washed in 0.15% Tween20 in TBS, and incubated for one hour with secondary antibody (1:10,000, Amersham Biosciences, New Jersey, United States) diluted in TBS. Anti-β-actin antibodies were used to control the loading. Proteins were visualized using SuperSignal chemiluminescent substrates (Thermo Scientific, Brookfield, Wisconsin, United States) and quantified using the FUJIFILM software (Fujifilm, http://www.fujifilm.com).

### Enzyme-linked immunosorbent assay (ELISA)

The amount of Tnf and Il1b in culture media was determined by using mouse Tnf-specific, and mouse Il1b-specific ELISA kits (eBioscience, San Diego, California, United States) according to the manufacturer’s protocols. The amount of Hsp70 in the vitreous humor was measured by using a mouse Hsp70-specific ELISA kit (Enzo Life Sciences, Farmingdale, New York, United States). The determination of the protein levels was performed according to the manufacturer’s protocols.

### Measurement of Hsp70 levels in the vitreous humor

It is known that the volume of vitreous humor in the mouse eye is 5.3 μl [29]. To collect the vitreous humor (VH), the sclera was penetrated by using a 33-gauge needle at a point temporal and posterior to the limbus, behind the lens. A small drop of VH that drained out was collected. This collected volume was equal to 2.5 μl, which was transferred in the tube containing 22.5 μl of PBS. Thus, the VH content was diluted tenfold. The tube was centrifuged at 1100 RPM for 15 minutes at +4°C and the supernatant was collected. To evaluate the Hsp70 level, ten microliters of the supernatant were added to 90 μl of ELISA buffer and the 100 μl were used for ELISA analysis. Therefore, the VH content was diluted tenfold a second time and the final dilution factor was 100. The measured Hsp70 levels were multiplied by 100, which gave us the final concentrations. To evaluate Hsp70 concentration in the GCL, we made the following assumptions: 1) GCL thickness is 30 μm, retinal area is 15.6 mm^2^[29], so the GCL volume (V_GCL_) was 468*10^−6^ ml; 2) only half of Hsp70 proteins released after IR are diffused in VH from GCL; 3) efficiency of Hsp70 penetration from the GCL to the VH due to the inner limiting membrane (the border between the VH and the retina) is 10 to 50% (1/10 ÷ 1/2).

### Intravitreal injection

Intravitreal injections were performed under a microsurgical microscope using glass pipettes with a diameter of approximately 150 μm at the tip. The left eye was punctured at the upper nasal limbus and a volume of 1 μl of Hsp70 (1 μg per injection) was injected. The contralateral eye was injected with a vehicle (control). To allow the solution to diffuse, the pipette was kept in place for about 15 seconds.

### Detection of endotoxins

All equipment, glassware, and materials used in the following procedures were either purchased as endotoxin-free or had been made endotoxin-free by heating at 250°C for 30 minutes. We took into account that the mean retinal thickness is 210 μm and retinal area is 15.6 mm^2^[29]. Thus, retinal volume (V_r_) was 3.276 μl. To measure endotoxin levels, 84 μl of endotoxin-free water was added to 20 retinas (66 μl). Then, three cycles of freezing in a liquid nitrogen bath and thawing in a 100°C water bath, followed by vortexing until tissue had dissolved as much as possible, were performed. The resulting tissue homogenates were centrifuged for 15 minutes at maximum speed and supernatants were collected. In order to evaluate the endotoxin level, 100 μl of the supernatant were used to measure the endotoxin by using the PYROGENT™ Ultra Gel Clot LAL (0.03 EU/ml sensitivity) assay, as recommended by the manufacturer (Lonza, Walkersville, Maryland, United States). To prepare serum, the blood was collected from the heart and allowed to clot by leaving it undisturbed at room temperature (30 to 60 minutes). The clot was removed by centrifuging at maximum speed for 15 minutes in a refrigerated centrifuge. The resulting supernatant (serum) was transferred into new endotoxin-free tubes and frozen at -80°C. All culture media were processed using a sterile technique and stored at -80°C prior to assays. The PYROGENT™ Ultra Gel Clot LAL (0.03 EU/ml sensitivity) assay was performed as recommended by the manufacturer (Lonza, Walkersville, Maryland, United States). To determine the endotoxin concentration, we tested serial two-fold dilutions of samples until an endpoint was reached.

### Statistical analysis

Statistical analysis was performed with a one-way analysis of variance (ANOVA) followed by the Tukey test for multiple comparisons. In the case of single comparisons, the Student’s t-test was applied. *P* values equal to or less than 0.05 were considered statistically significant.

## Results

### Polymyxin B mediates neuroprotection after ischemia-reperfusion injury due to protein kinase C inhibition rather than endotoxin inhibition

**Figure 1:**
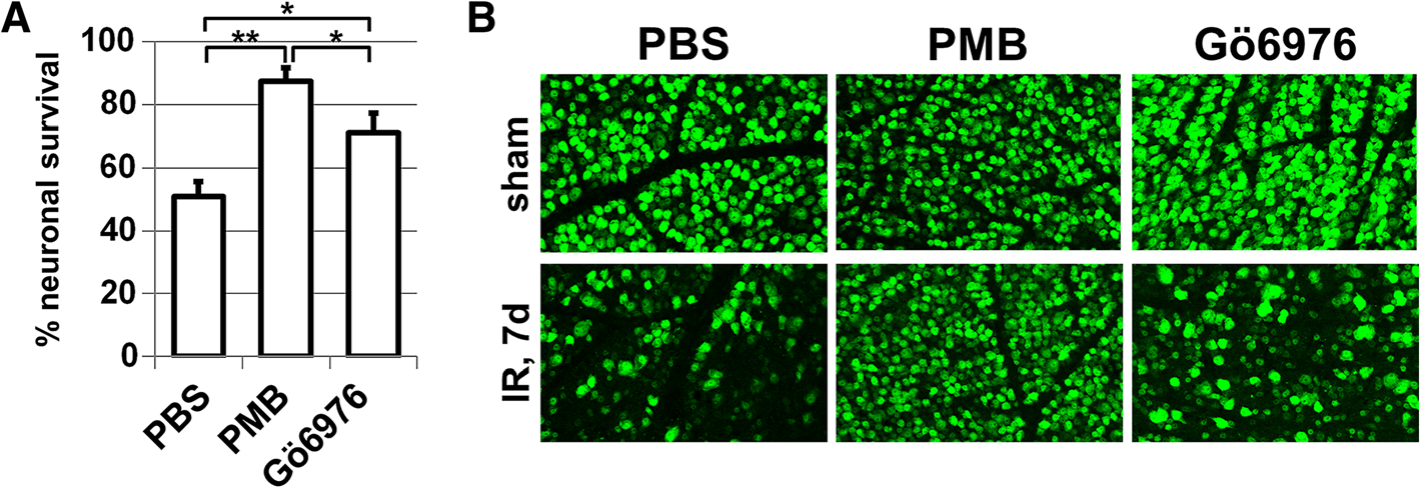
**Treatment with PMB and Gö6976 results in neuroprotective effects in the GCL after IR: A)** Percentage of GCL neurons lost at one week following IR of C57bl/6 mice treated with PMB, Gö6976, and PBS (control) (**P < 0.01, *P < 0.05, n = 5-7). **B)** Representative confocal images of NeuN-labeled GCL (green) neurons in flat-mounted retinas in sham-operated controls and ischemic retinas 7 days after reperfusion. GCL, ganglion cell layer; IR, ischemia reperfusion; NeuN, neuronal nuclei; PBS, phosphate buffered saline; PMB, polymyxin B.

**Figure 2:**
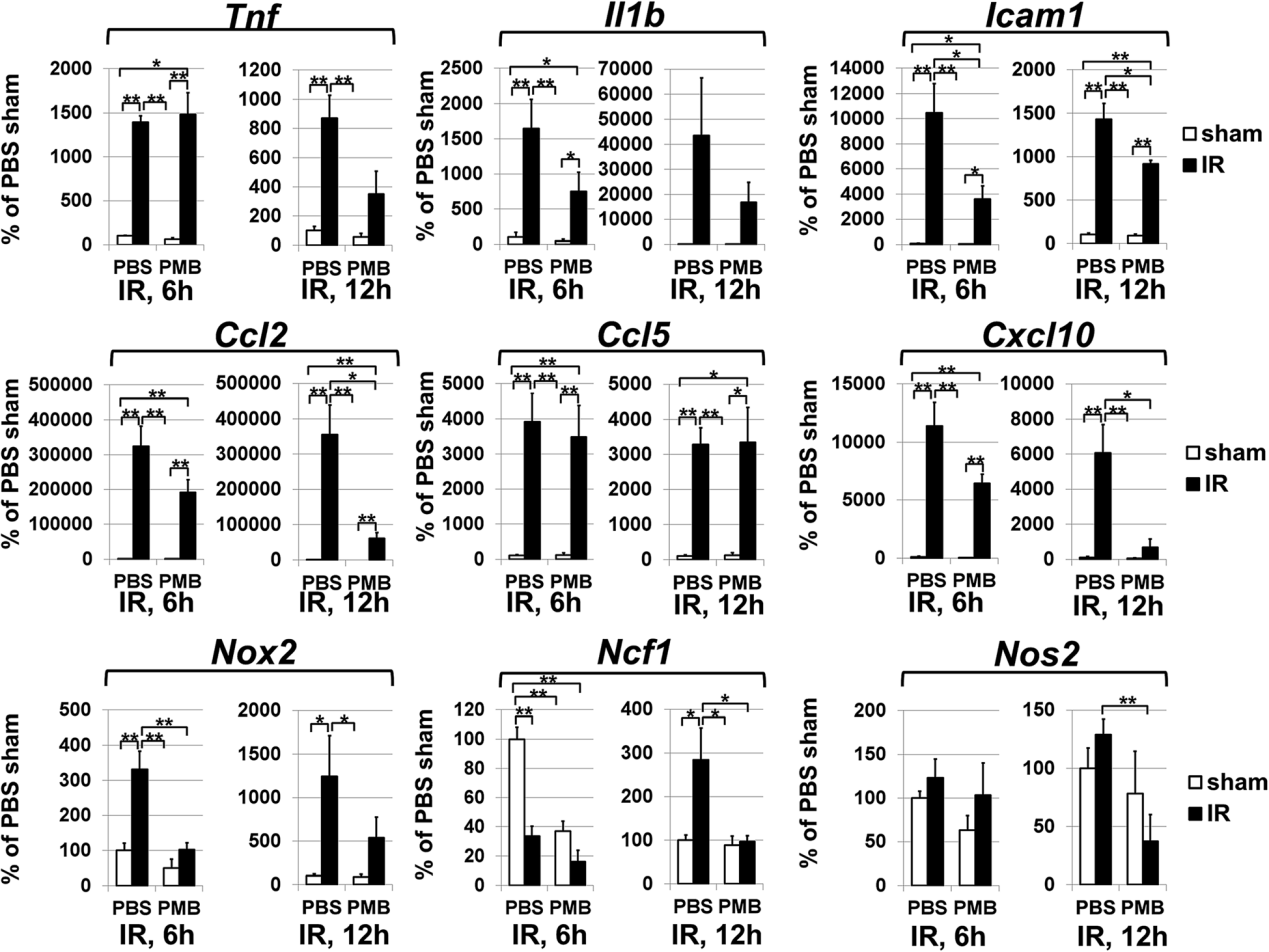
**PMB treatment reduces pro-inflammatory gene expression following IR injury.** Differential expression of cytokines, chemokines, cell adhesion molecules, as well as genes encoding *Nos2* and subunits of the reactive oxygen species-producing NAD(P)H oxidase was assessed in sham-operated and ischemic retinas of PMB- and PBS-treated animals, 6 and 12 hours after reperfusion, by using quantitative RT-PCR. For each gene, results are expressed as a percentage of corresponding value in the sham-operated eye of PBS-treated animals (*P < 0.05, **P < 0.01, five animals per group). IR, ischemia reperfusion; PBS, phosphate buffered saline; PMB, polymyxin B.

Necrosis is a pathologic hallmark of IR injury in the CNS [4-7]. Retinal IR injury results in a prolonged period of cell death with a high level of necrosis at the early stage of pathology [4,5,30]. We have previously demonstrated that the release of DAMPs, as a result of retinal necrosis and Tlr4 signaling, contributes to IR-induced retinal injury [5,8,11]. Thus, the retinal ischemia model was the most suitable model to achieve the objectives of this study. We first asked whether endotoxins play a role in IR-induced retinal injury. To answer this question, we used PMB, a known inhibitor of endotoxin activity [22]. Mice were injected IP with PMB (8 μg/g body of mouse or 7.5 μM) or PBS (control) one hour before IR, six hours after surgery, and twice every twenty four hours until they were euthanized seven days later, after reperfusion. Whole retina flat-mounts were stained for the neuronal marker NeuN to quantify the number of surviving neurons in the GCL. We found that retinas from the experimental eyes of PBS-treated mice had significantly lower numbers of surviving NeuN-positive neurons in the GCL compared to PMB-treated mice (51 ± 5% versus 87 ± 4%, *P* < 0.01) (Figure 1). To evaluate the molecular changes associated with resistance to IR injury in PMB-treated retinas, we investigated the activation of several pro-inflammatory markers known to be involved in IR-induced cytotoxicity. We compared gene expression in ischemic versus sham-operated eyes for both PBS- and PMB-treated mice. We found transcriptional up-regulation of cytokines (*Tnf*, *Il1b)*, chemokines (*Ccl2*, *Ccl5*, and *Cxcl10),* cell adhesion molecule (*Icam1)*, as well as genes encoding subunits of the reactive oxygen species-producing NAD(P)H oxidase, in all experimental eyes at both 6 and 12 hours after reperfusion (Figure 2). In PMB-treated mice, however, the expression of *Tnf*, *Il1b*, *Ccl2*, *Cxcl10*, *Icam1*, *Ccl2*, *Cxcl10*, and subunits of NAD(P)H oxidase *Cybb* (*Nox2*) and *Ncf2* was reduced relative to PBS-treated mice (Figure 2). One interpretation of these data is that PMB promotes neuroprotection by reducing pro-inflammatory activity in ischemic tissue.

The next step in our study was to detect endotoxin levels in the retina. To evaluate the endotoxin levels in the retina, we used a gel clot LAL assay kit with sensitivity of 0.03 EU/ml (since 1 EU = 100 pg, then 0.03 EU/ml = 3 pg/ml). We collected and analyzed endotoxin levels in three groups containing 20 retinas each. Surprisingly, we did not detect a positive reaction for any of the three groups using the gel clot LAL assay. Since the presence of endotoxins in the blood can affect endotoxin levels in the retina after IR due to blood-retinal barrier breakdown, we also attempted to determine the level of endotoxins in serum, but did not find detectable levels of circulating endotoxins. Given that PMB functions by inhibiting endotoxins and the fact that we did not detect endotoxins, these data suggested that the observed PMB effect in ischemic tissue was probably not due to endotoxin presence. To explain the PMB effect in absence of endotoxins, we noted that PMB is also a PKC inhibitor (IC50, 2-10 μM) [23-25]. Our calculations, presented in the ‘Methods’ section, indicated that the concentration of PMB in the retina was at least 7.5 μM. Thus, we asked whether or not PMB’s neuroprotection could be due to PKC inhibition in ischemic tissue. To test this hypothesis, we used a PKC inhibitor (Gö6976), which selectively inhibits Ca^2+^-dependent PKC isozymes similar to PMB. The animals were treated with Gö6976 (167 ng/g body of mouse or 551 nM) IP one hour before ischemia, six hours after surgery, and twice every twenty four hours until they were euthanized. Control animals were treated with PBS. After seven days, the retinas were collected and stained for the neuronal marker NeuN, and the percentage of surviving GCL neurons was revealed. We found that the percentage of surviving GCL neurons in the IR retinas was significantly higher in mice injected with Gö6976 (71 ± 6%) compared with those injected with PBS (51 ± 5%, *P* < 0.05) (Figure 1). Collectively, these data suggest that perhaps the observed PMB-induced protection was due to PKC inhibition, rather than its endotoxin inhibiting function.

### Presence of protein kinase C but not endotoxins critical to mediate Hsp70-dependent pro-inflammatory response

**Figure 3:**
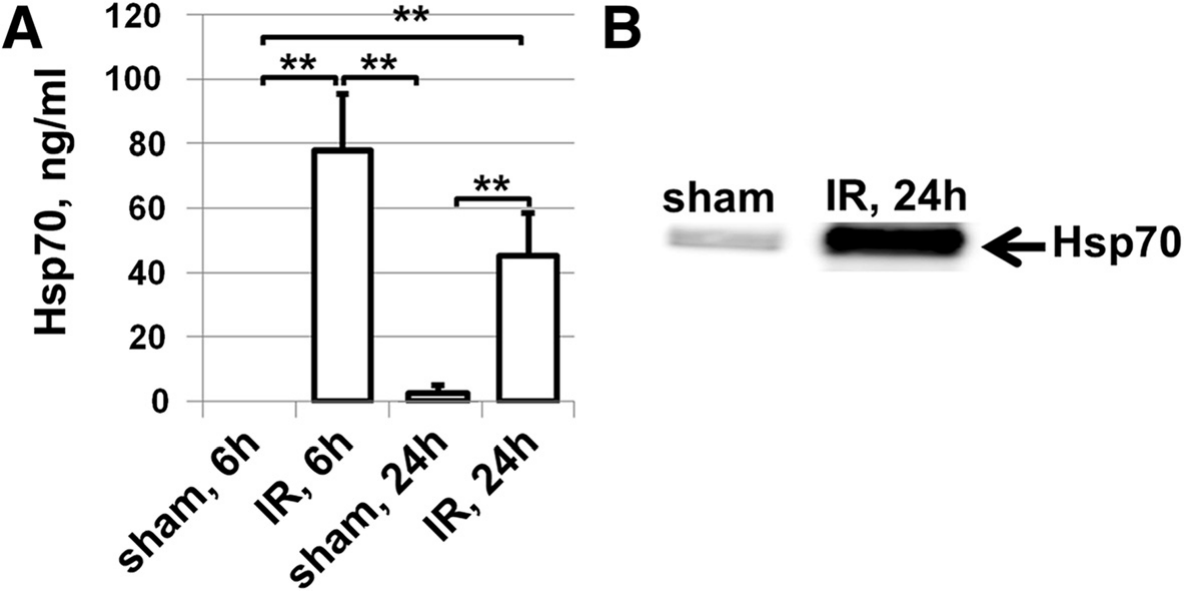
**Extracellular Hsp70 is accumulated in the vitreous humor of animals suffering from retinal ischemia: A)** Level of Hsp70 in vitreous humor of sham-operated and ischemic eyes 6 and 24 hours after reperfusion. **B)** Representative western blot. h, hours; Hsp70, heat shock protein 70; IR, ischemic reperfusion.

**Figure 4:**
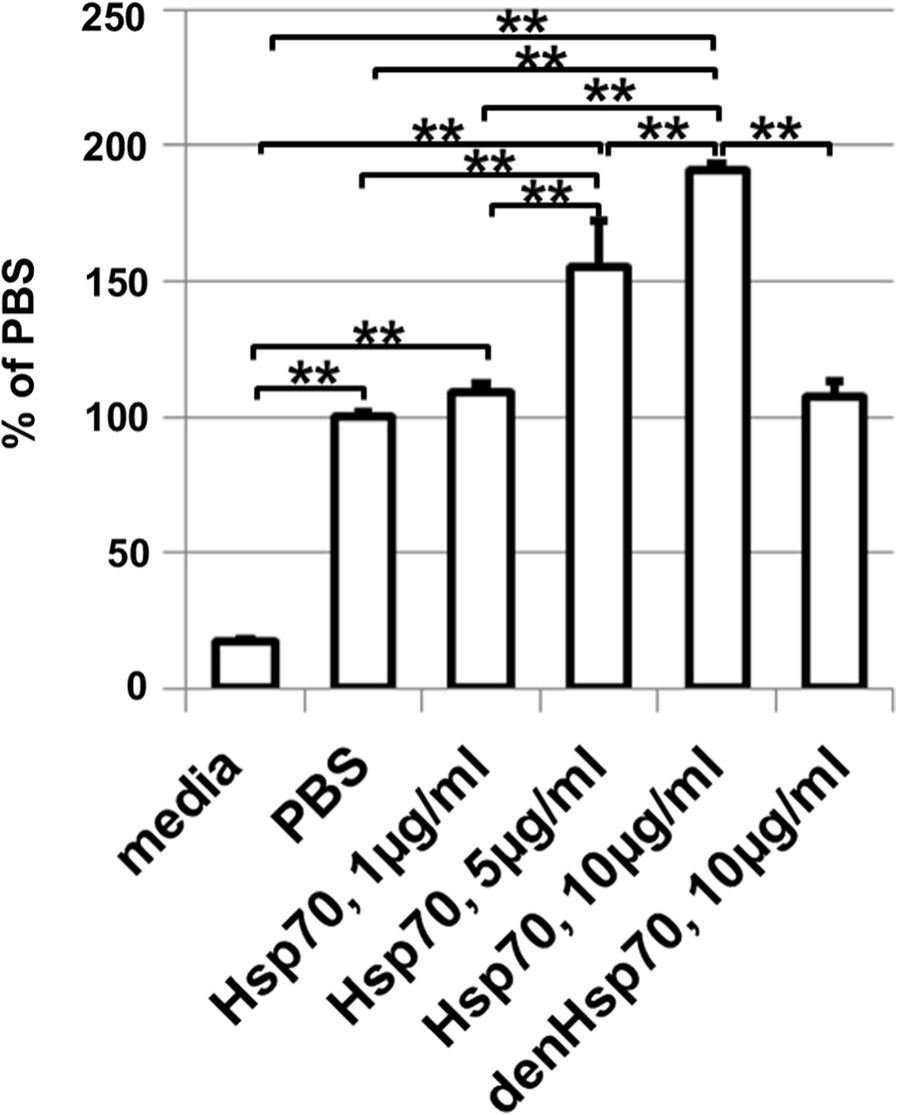
**Stimulation of RAW-blue reporter cell line with Hsp70 leads to an increase in NF-κB activation.** denHsp70 samples were heated for one hour at 56°C before treatment of cell cultures. Hsp70, heat shock protein 70; PBS, phosphate buffered saline.

To study in detail the observed effects in our model, we needed to identify Tlr4 ligands, which can initiate a pro-inflammatory response. Since Hsp70 is a Tlr4 ligand that can stimulate Tlr4 downstream pro-inflammatory effects, we tested whether Hsp70 is released in the extracellular space after IR injury. We measured Hsp70 levels in the VH of the eye 6 and 24 hours after IR injury in the retina by using the mouse Hsp70-specific ELISA kit (Figure 3). We observed an increase in Hsp70 protein content in the VH of ischemic eyes 6 hours (78 ± 17 ng/ml) and 24 hours (45 ± 13 ng/ml) after reperfusion compared to sham-operated controls (0 ± 0 ng/ml and 2 ± 2 ng/ml). Thus, our data indicated that Hsp70 concentration in the VH of ischemic eyes was in the range of 50 to 100 ng/ml. Since VH volume is approximately 5.3 μl in mice [29], the quantity of Hsp70 in the VH is estimated to be 265 to 530 pg. Since only half of Hsp70 proteins are diffused in the VH from the GCL, the GCL may contain 530 to 1060 pg of Hsp70. However, since there is the inner limiting membrane between the VH and the GCL that poses a barrier for penetration of Hsp70 into the VH from the GCL, the actual quantity of the extracellular Hsp70 in the GCL should be in the range of 1-10 ng (Methods). Since GCL volume is 468*10^−6^ ml (Methods), the real concentration of Hsp70 in GCL after IR should be in the range of 2 ÷ 20 μg/ml. Based on these data, we evaluated the minimum concentration of Hsp70 required to initiate a pro-inflammatory response. To figure this out, we used a mouse macrophage RAW-blue reporter cell line which, upon Tlr4 stimulation, initiates NF-κB-dependent production and secretion of easily detectable and measurable embryonic alkaline phosphatase (SEAP). The RAW-blue cells were treated with recombinant Hsp70 protein. When we first measured endotoxin levels in culture media containing the recombinant Hsp70, we found that 10 μg/ml of Hsp70 contains 3 pg/ml (0.03 EU/ml) of endotoxins. Measuring the level of SEAP in the media of Hsp70 treated RAW-blue cells, we have detected that the combination of 5 μg/ml of Hsp70 and 1.5 pg/ml (0.015 EU/ml) of endotoxins is enough to activate NF-κB (Figure 4). We also found that heat inactivation of Hsp70 significantly reduced SEAP production in RAW-blue cells.

**Figure 5:**
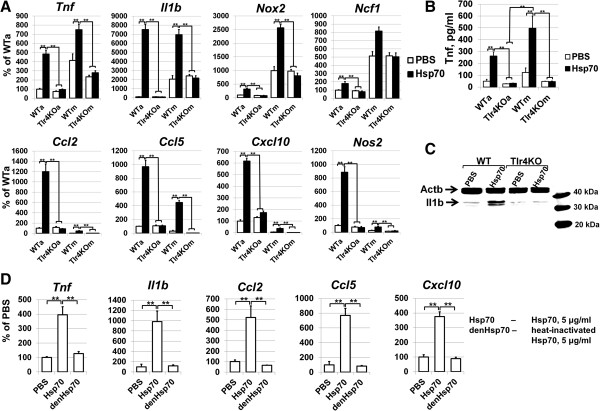
**Hsp70 mediates production of pro-inflammatory factors in a Tlr4-dependent manner. A)** Differential expression of cytokines, chemokines, as well as genes encoding Nos2 and subunits of the reactive oxygen species-producing NAD(P)H oxidase was assessed in primary cultures of astrocytes and microglia isolated from wild type (WTa and WTm referred to WT astrocytes and WT microglia) and Tlr4KO animals (Tlr4KOa and Tlr4KOm referred to Tlr4KO astrocytes and Tlr4KO microglia), and treated with Hsp70 (5 μg/ml) containing 1.5 pg/ml of endotoxins and PBS (control) during a 24-hour period. For each gene, results were expressed as a percentage of the corresponding value in the wild type astrocytes (WTa) treated with PBS (**P < 0.01, n = 6). **B)** The amount of Tnf in culture media from astrocytes and microglia isolated from wild type and Tlr4KO mice and treated with Hsp70 were determined by using mouse Tnf-specific ELISA kits. (**P < 0.01, n = 6). **C)** Hsp70 induces production of the immature form of Il1b, but not the active form of Il1b in astrocytes. The astrocytes isolated from wild type and Tlr4KO animals were treated with Hsp70 (5 μg/ml) and lysed after 24 hours. Forms of Il1b were analyzed by western blot analysis. **D)** Heat inactivation of recombinant Hsp70 significantly reduced pro-inflammatory response of treated primary astrocytes. Primary astrocytes were treated for 24 hours with recombinant Hsp70 (5 μg/ml), heat-inactivated recombinant Hsp70 (5 μg/ml), and PBS as a control (**P < 0.01, n = 6). For each gene, results are expressed as a percentage of corresponding value in the astrocytes treated with PBS (control) (denHsp70 samples were heated for one hour at 56°C before treatment of cell cultures). Hsp70, heat shock protein 70; PBS, phosphate buffered saline; WT, wild type.

**Figure 6:**
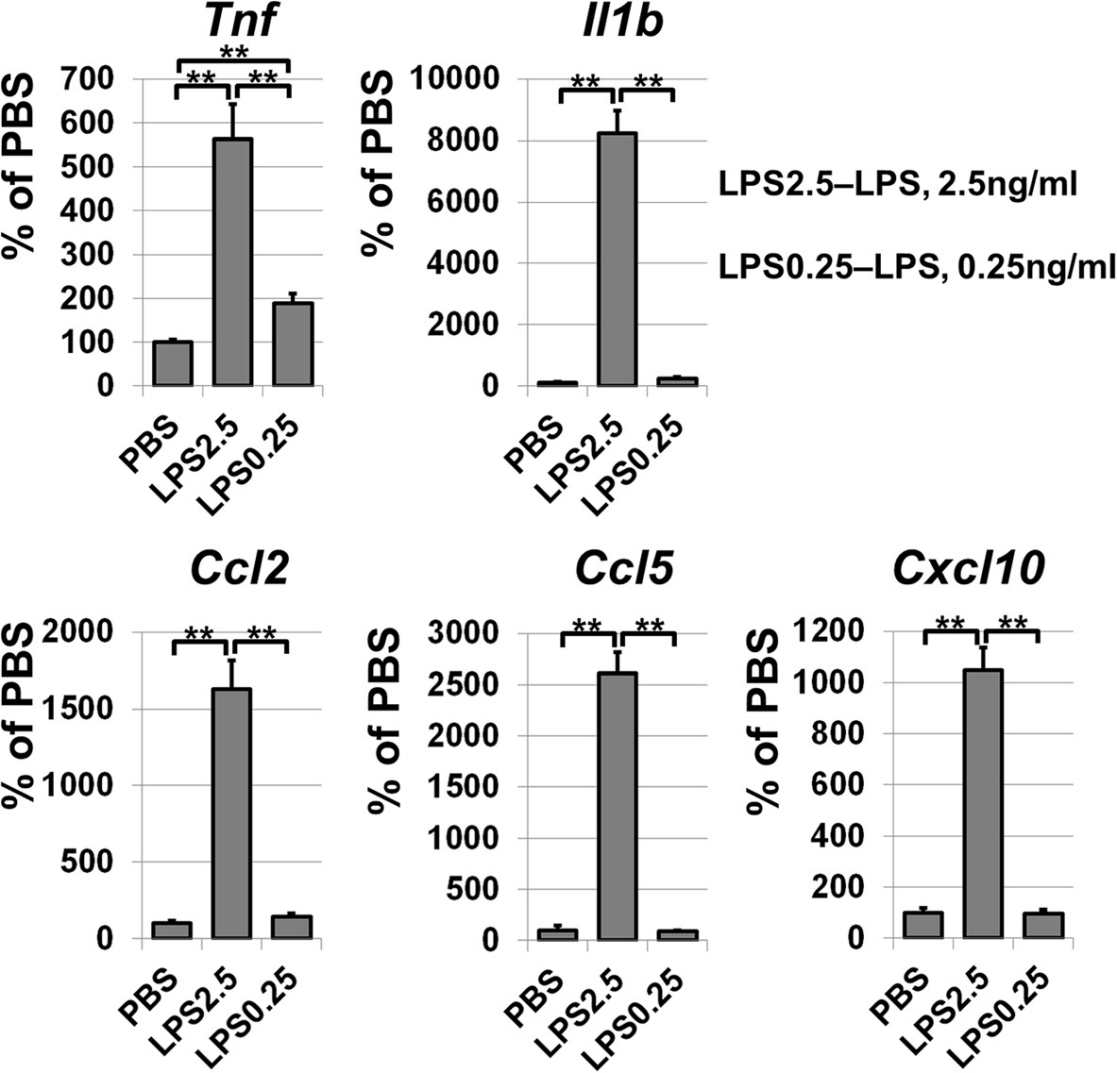
**Astrocytes respond differently to varying concentrations of LPS.** Stimulation of primary astrocytes with 2.5 ng/ml of LPS leads to increased expression of pro-inflammatory genes, while the pro-inflammatory response of primary astrocytes treated with 0.25 ng/ml was insignificant. Gene expression was assessed in primary astrocytes treated 24 hours with LPS and PBS (controls) by quantitative RT-PCR. For each gene, results were expressed as a percentage of the corresponding value in the astrocytes treated with PBS. LPS, lipopolysaccharides; PBS, phosphate buffered saline.

Next, we investigated the Hsp70-dependent pro-inflammatory response of glial cells, because their activation was shown to occur temporally before leukocyte infiltration in ischemic retina. Glial cells (astrocytes and microglia) were isolated from WT and Tlr4 knockout animals (Tlr4KO) and treated for 24 hours with 5 μg/ml of Hsp70 containing 1.5 pg/ml (0.015 EU/ml) of endotoxins. We observed increased levels of cytokine, chemokine, NAD(P)H oxidase, and nitric oxide synthase activity in Hsp70-treated glial cells compared to the PBS-treated control (Figure 5). This activity was significantly reduced in glial cells isolated from Tlr4KO animals (Figure 5) as well as in glial cells treated with heat-inactivated Hsp70 (Figure 5). Next we asked the question of whether such low detected levels of endotoxins can alone initiate a pro-inflammatory response. To answer this question, we tested the pro-inflammatory activation of glial cells at different concentrations of LPS. Since we observed a similar pro-inflammatory response of astrocytes and microglial cells upon Hsp70 treatment, the experiments were carried out on primary astrocytes isolated from wild type animals. We found that 2.5 ng/ml of LPS mediated pro-inflammatory activation of astrocytes, while 250 pg/ml of LPS had no stimulatory effects upon astrocytes (Figure 6). Thus, detected low endotoxin levels either play no role or can only amplify Hsp70 pro-inflammatory effects.

**Figure 7:**
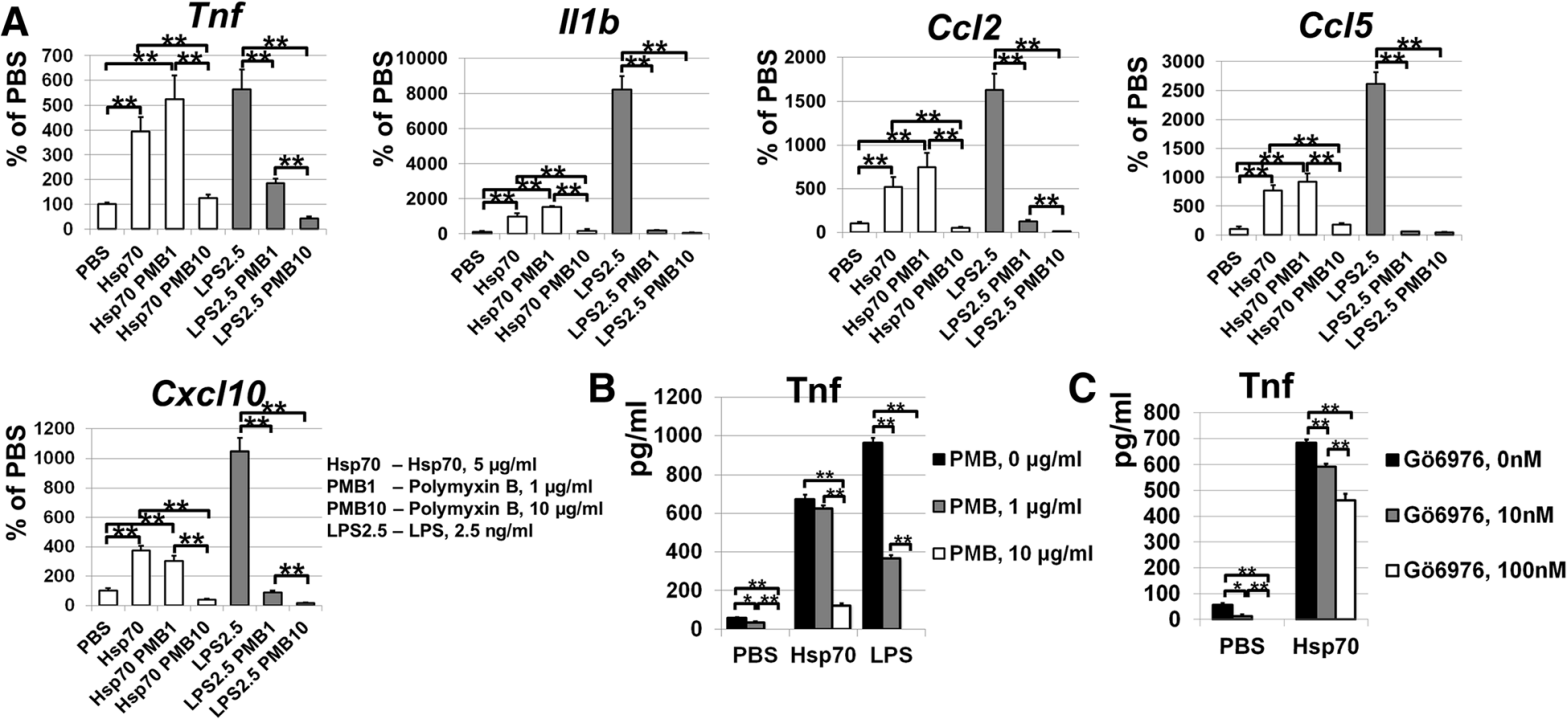
**PMB in Hsp70-treated astrocytes behaves as PKC inhibitor rather than endotoxin inhibitor. A)** Pretreatment of astrocytes only with the highest PMB concentration (10 μg/ml) caused significant reductions in the pro-inflammatory activity induced by the recombinant Hsp70, while the pro-inflammatory response of LPS-treated astrocytes was significantly reduced by the addition of 1 and 10 μg/ml of PMB. Primary astrocytes were treated with Hsp70 (5 μg/ml) containing 1.5 pg/ml of endotoxins or LPS (2.5 ng/ml) in the presence or absence of PMB (**P < 0.01, n = 6). Differential expression of cytokines and chemokines was assessed in primary astrocytes after 24 hours by using quantitative RT-PCR. **B)** The amounts of Tnf in culture media from astrocytes treated with Hsp70 (5 μg/ml) or LPS (2.5 ng/ml) in the presence or absence of PMB were measured by using ELISA (**P < 0.01, *P < 0.05, n = 6). **C)** The level of Tnf was significantly reduced in media from astrocyte cultures treated with Hsp70 in the presence or absence of PKC inhibitor (Gö6976, 10 nM and 100 nM). Culture media was collected 24 hours after treatment, and then the Tnf level was detected using the ELISA kit (**P < 0.01, n = 6). Hsp70, heat shock protein 70; LPS, lipopolysaccharides PKC, protein kinase C; PMB, polymyxin B.

To answer the question of whether the detected low endotoxin levels affect pro-inflammatory activity of Hsp70, we performed an additional series of experiments using PMB. The primary astrocytes were treated 24 hours with 5 μg/ml of Hsp70 containing 1.5 pg/ml of endotoxins, LPS (2.5 ng/ml) in the presence or absence of PMB (1 and 10 μg/ml). The cells were lysed after 24 hours and transcriptional levels of cytokines and chemokines were analyzed using quantitative PCR. We also tested the Tnf level in the media of treated cells using a mouse Tnf-specific ELISA kit. We found that the pro-inflammatory response of LPS-treated astrocytes was significantly reduced by the addition of 1 μg/ml (0.7 μM) of PMB (Figure 7A). At the same time, Hsp70–treated astrocytes in the presence of 1 μg/ml (0.7 μM) of PMB did not reduce but rather increased expression of the cytokines and chemokines (Figure 7A). We also found that the level of Tnf in culture media from LPS/PMB-treated cells was significantly reduced, while the level of Tnf in culture media from Hsp70/PMB-treated cells was just slightly/insignificantly reduced (Figure 7B). However, treatment of cell cultures with 10 μg/ml (7 μM) of PMB significantly reduced expression levels of tested cytokines and chemokines in astrocytes mediated by Hsp70 and LPS (Figure 7A). Interestingly, we were still able to detect Tnf in media from PMB- (10 μg/ml or 7 μM) and Hsp70-treated cells, while presence of Tnf in media from PMB- (10 μg/ml or 7 μM) and LPS-treated cells was not detected (Figure 7B). It should be recalled that PMB inhibits PKC with an IC50 equal to 2-10 μM [23-25]. Thus, Hsp70-activated cells behave as if PMB inhibits only PKC and the presence of low levels of endotoxins does not play a role, while LPS-activated cells behave as if PMB inhibits LPS and PKC. It was shown previously that PKC activation is critical in LPS-mediated production of pro-inflammatory factors by macrophages [26]. To test the role of PKC in Hsp70-dependent pro-inflammatory response, astrocytes were treated with Hsp70 in the presence or absence of PKC inhibitor (Gö6976, 10 nM and 100 nM). After 24 hours, culture media was collected and the Tnf level was detected using the ELISA kit. We found that the level of Tnf was significantly reduced in Gö6976-treated cells (Figure 7C). Collectively, these data suggest that PKC is critical to mediate the Hsp70-dependent pro-inflammatory response. At the same time, the presence of low endotoxin levels in Hsp70 preparations is not critical in mediating the production of pro-inflammatory factors. Importantly, PMB in Hsp70-treated cultures behaves as a PKC inhibitor.

### Extracellular Hsp70 mediates Tnf-dependent neuronal death

**Figure 8:**
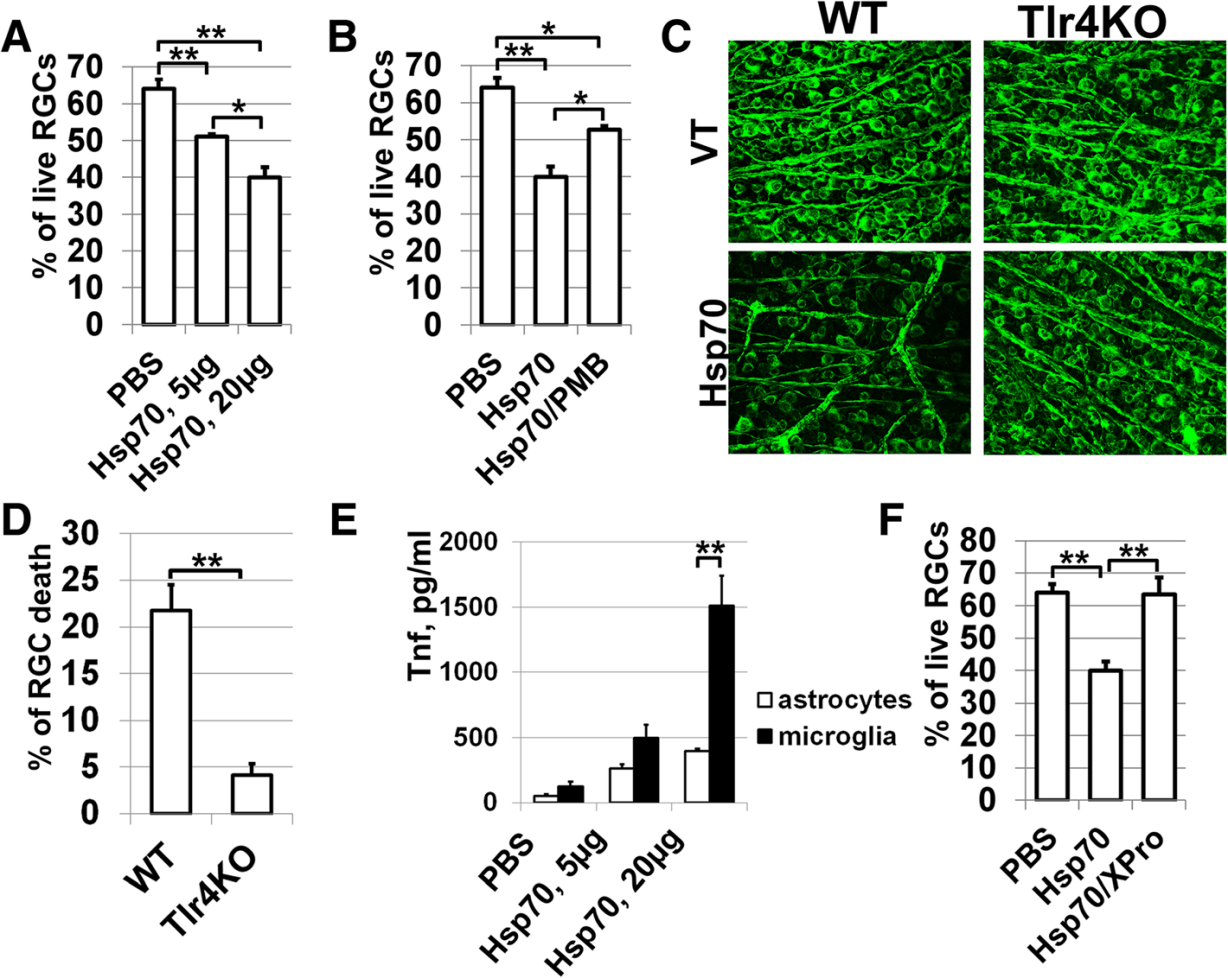
**Extracellular Hsp70 promotes RGC death by mediating production of cytotoxic levels of Tnf by glial cells. A)** Recombinant Hsp70 contributes to RGC death *in vitro.* RGC/glia co-cultures were treated 24 hours with 5 and 20 μg/ml of recombinant Hsp70, and RGC death was detected by using the Apoptosis Assay Kit #2; (Invitrogen, United States). **B)** Inhibition of PKC activity in RGC/glia co-cultures with PMB helps to protect RGCs from neurotoxicity mediated by Hsp70. RGC/glia co-cultures were treated 24 hours with 20 μg/ml of Hsp70 in the presence of 10 μg/ml of PMB or PBS (control) and then RGC death was detected. **C)** Representative confocal images of beta III Tubulin-labeled RGCs in the GCLs (green) of flat-mounted retinas of eyes intravitreally injected with Hsp70 (1 μg per injection) and control eyes (vehicle treated; VT) were obtained seven days after treatment. **D)** The percent of RGC death in control and Hsp70-treated eyes of WT and Tlr4KO animals (**P < 0.01, n = 6). **E)** Glial cells treated with Hsp70 produce sufficient amounts of Tnf to initiate neuronal death. **F)** Treatment with Hsp70 increased RGC death in the RGC/glia co-cultures, while treatment with Hsp70 in the presence of Tnf inhibitor (XPro1595) resulted in RGC protection. Hsp70, heat shock protein 70; PMB, polymyxin B, PKC, protein kinase C; RGC, retinal ganglion cells.

Changes in the levels of intracellular and extracellular Hsp70 can produce different responses after IR injury: intracellular Hsp70 mediates neuroprotection after IR [31,32], while our results indicate that extracellular Hsp70, providing that PKC is activated, should induce a strong neurotoxic pro-inflammatory response in tissue. Thus, interplay between pro-survival and death-promoting signaling cascades can continue until one eventually dominates and determines the cell’s fate. We propose that a high level of extracellular Hsp70 released as a result of ischemic stress initiates a severe neurotoxic pro-inflammatory response, which far exceeds Hsp70 neuroprotective activity and finally mediates neuronal death. To test this hypothesis, we first determined the concentration of Hsp70, which can mediate RGC death in RGC/glia co-culture. Primary RGCs were plated on cover slips in a 24-well plate and glial cells (50% primary astrocytes and 50% microglial cells) plated in culture inserts were placed into the culture wells containing RGCs prior to Hsp70 treatment. These co-cultures were treated with 5 μg/ml and 20 μg/ml of Hsp70. The level of RGC death was assessed after 24 hours. Quantification of RGC death showed significantly higher cell death in cultures treated with Hsp70 than with PBS (Figure 8A). However, when RGC loss was compared between cultures treated with 5 and 20 μg/ml of Hsp70, significant differences in neuronal viability became apparent. RGCs treated with 20 μg/ml of Hsp70 had lower numbers of RGC survival (40 ± 3%) than RGCs treated with 5 μg/ml of Hsp70 (51 ± 1%, *P* < 0.05, Figure 8A). To evaluate the contribution of PKC activity in the observed Hsp70-dependent RGC death, we used RGC/glia co-culture treated with 20 μg/ml of Hsp70 in the presence of 10 μg/ml (7 μM) of PMB. Co-cultures treated with Hsp70 only were used as controls. We found that RGC cultures in the presence of a PKC inhibitor were protected against treatment with Hsp70 and demonstrated a reduced level of RGC death (53 ± 1% vs 40 ± 3%, *P* < 0.05) (Figure 8B). These *in vitro* data suggest that extracellular Hsp70 released as a result of ischemic stress can mediate neuronal death if there is PKC activity in glial cells. To verify the observed results *in vivo*, the left eyes of WT and Tlr4KO mice were intravitreally injected with Hsp70. The contralateral eyes were treated with a vehicle and used as controls. The retinas were collected seven days after treatment and whole retina flat-mounts were stained for the RGC marker beta III Tubulin to quantify the number of surviving RGCs in the GCL (Figure 8C). We observed that retinas from experimental eyes of WT mice had a significantly higher percentage of dead RGCs (21 ± 3%) than the Tlr4KO mice (4 ± 1%, *P* < 0.01, Figure 8D). To evaluate the mechanism of Hsp70-mediated RGC death, we noted that treatment of glial cells with Hsp70 induced significant Tnf production (Figure 8E). It was shown previously that Tnf plays a largely deleterious role in retinal IR injury [33]. To study the role of Tnf in Hsp70-mediated RGC death, RGC/glia co-cultures were challenged with 20 μg/ml of Hsp70 in the presence or absence of XPro1595, a selective soluble Tnf blocker. Levels of RGC survival in these co-cultures were assessed 24 hours after treatment. We found that treatment with Hsp70 only decreased the number of live RGCs (40 ± 3%), while the RGC death was reduced in the presence of XPro1595 (64 ± 5%, *P* < 0.01) (Figure 8F). Thus, one way that extracellular Hsp70 can promote neuronal death is by mediating the production of cytotoxic levels of Tnf by glial cells.

**Figure 9:**
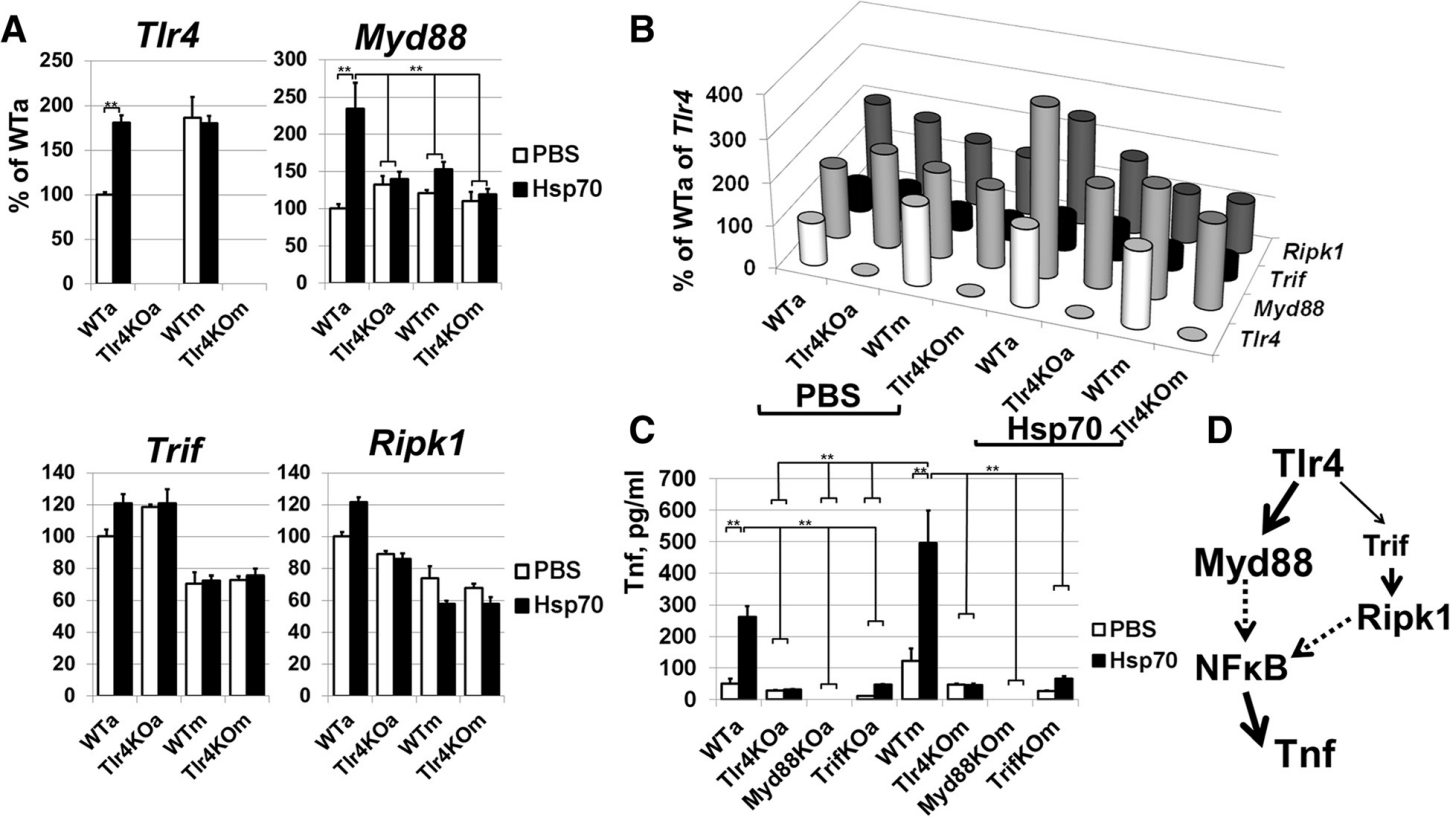
**Upon Hsp70 engagement, Tlr4 initiates a pro-inflammatory response via Myd88- and Trif-dependent signaling cascades. A)** Expression analysis of Tlr4 signaling cascade genes in Hsp70 (5 μg/ml) and PBS-treated glial cells demonstrated increased expression of *Myd88* in wild type astrocytes (**P < 0.01, n = 6; WTa and WTm referred to WT astrocytes and WT microglia; Tlr4KOa and Tlr4KOm referred to Tlr4KO astrocytes and Tlr4KO microglia). **B)** A comparative analysis of *Tlr4, Myd88, Trif, Ripk1* gene expression in wild type and Tlr4KO glial cells suggests the preferential activation of Myd88 signaling cascade. **C)** The amount of Tnf in culture media from wild type, Tlr4KO, Myd88KO and TrifKO glial cells treated with Hsp70 and PBS (control) (**P < 0.01, n = 6). **D)** Two main downstream branches of Tlr4 signaling. Hsp70, heat shock protein 70; PBS, phosphate buffering saline; WT, wild type.

Since Tlr4 signaling consists of two distinct signaling cascades, Myd88- and Trif/Ripk1-dependent [10,34,35], we evaluated the expression of *Tlr4*, *Myd88*, *Trif* and *Ripk1* in primary glial cells isolated from WT and Tlr4KO animals and treated with 5 μg/ml of Hsp70 to identify cascades involved in Hsp70-dependent neurotoxicity. We found that the transcriptional levels of *Tlr4* and *Myd88* were increased in wild type astrocytes treated with Hsp70, but not in microglia (Figure 9A). We also found no significant changes in the expression of *Trif* and *Ripk1* (Figure 9A). However, whereas astrocytes and microglia expressed only a low level of *Trif*, they exhibited significantly higher levels of *Myd88* (Figure 9B). These data suggest that the role of the Myd88 signaling cascade may be more important in Hsp70-dependent pro-inflammatory activation of glial cells than the role of the Trif signaling cascade. To test this hypothesis, primary glial cells were isolated from WT, Tlr4, Myd88- and Trif-deficient animals, treated with Hsp70 as described above, and then the Tnf levels were measured in the media of these glial cells. We detected significantly reduced levels of Tnf in media of Hsp70-treated Tlr4, Myd88- and Trif-deficient glial cell cultures compared to glial cells isolated from WT animals (Figure 9C). However, the level of Tnf in media of Hsp70-treated Myd88KO glial cells was reduced significantly compared to TrifKO glial cells treated with Hsp70 (Figure 9C), which supports the hypothesis.

## Discussion

Sterile inflammation, or innate immune response in the absence of live pathogens, is an unavoidable consequence of IR injury in the CNS, which jeopardizes the viability of the neurons at the site of injury [1-3]. It has been shown that IR-induced degenerative conditions can be significantly improved by modulating the activity of pro-inflammatory signaling cascades [1,36]. However, it would be more effective to eliminate the triggers of sterile inflammation rather than blocking their effector pathways. Many results obtained *in vivo* indicate that the triggers of sterile inflammation are DAMPs, which are released outside the cell following tissue injury, such as injury induced by IR, and the subsequent activation of pattern recognition receptors [8,9]. Tlr4 is a pattern recognition receptor that initiates an innate immune response in the presence of bacteria-derived endotoxins and DAMPs [9,10]. Importantly, the critical role of Tlr4 was shown in damage and sterile inflammation triggered by IR in the CNS [11,12,37]. However, *in vitro* studies suggest that DAMPs alone cannot activate Tlr4, and thus additional factors should be involved in the DAMP-mediated pro-inflammatory response in tissue. In our study, we focused on extracellular Hsp70, a prototypic DAMP molecule and ligand of Tlr4, because it was released at high levels after IR in the retina. We tested whether the presence of low endotoxin levels (subclinical level, not leading to pro-inflammatory response) in ischemic tissue or PKC activity were involved in Hsp70-mediated pro-inflammatory neurotoxicity. Since activation of glial cells was shown to occur temporally before leukocyte infiltration in ischemic tissue, we used primary astrocyte and microglia cell cultures in our study. We found that extracellular Hsp70 activates the Tlr4 signaling cascade in glial cells that initiate the production of neurotoxic factors. PKC activity was critical to initiate the Hsp70-dependent pro-inflammatory response. Meanwhile, the low endotoxin levels were not involved in Hsp70-mediated pro-inflammatory activity.

The concept that endotoxins can be involved in DAMP-mediated pro-inflammatory activity became evident when we noted that: 1) the presence of low endotoxin levels was shown in human and animal tissues [18-21,38]; and 2) endotoxins, even in small amounts, act in synergy and amplify the DAMP-mediated pro-inflammatory response [9,15-17,39]. It was suggested that the increased endotoxin levels in tissues might be a result of endotoxin translocation derived from the resident bacteria in the gastrointestinal tract [40-42]. However, detected endotoxin levels were too low to have any pathological significance in healthy tissue. At the same time, the low endotoxin levels dramatically sensitize the immature brain to injury and induce cerebral infarction in response to short periods of hypoxia-ischemia [43]. In addition, an endotoxin inhibitor can block heat exposure-induced expression of brain cytokines [18]. Thus, DAMPs released as a result of pathological conditions, such as IR, combined with a low-dose endotoxin in the tissue could initiate a pro-inflammatory response that promotes cell death and tissue damage. Since it was shown in some studies that pure (endotoxin free) DAMPs could not mediate a pro-inflammatory response, the concept received our considerable attention. Like many others, we used the endotoxin inhibitor, PMB, in our study [22]. We found significant neuroprotection and a reduced pro-inflammatory response in ischemic retinas as a result of PMB treatment. However, we did not detect any endotoxin presence in the studied tissues. We also noted that in the cited papers, endotoxin levels were within the sensitivity range of the assay used. In addition, Rivera *et al*. [21] detected endotoxins in the portal vein but not the vena cava, showing endotoxins to be present in blood coming from the gastrointestinal tract to the liver, and then being detoxified in the liver before being distributed to the rest of the body. It is therefore hard to conclude whether the neuroprotective effect of PMB *in vivo* is due to inhibition of undetectable levels of endotoxin, or other effects that are unrelated to endotoxins. PMB is also a PKC inhibitor [23-25], and thus, the *in vivo* protective effects could be due to inhibition of PKC. To test this hypothesis we treated the animals with a known PKC inhibitor (Gö6976) and found significant neuroprotection. Therefore, the neuroprotective effect in PMB-treated ischemic retinas should be due to PKC inhibition rather than endotoxin inhibition.

The universally expressed PKC family in vertebrate tissues is involved in many signaling cascades, including the innate immune response [26,44]. PKC isozymes modify other proteins by chemically adding phosphate groups to them from a nucleoside triphosphate (ATP) and are classified as conventional, novel and atypical [26,44,45]. Conventional PKC isozymes are Ca^2+^-dependent, while novel and atypical isozymes do not require Ca^2+^ for their activation. In our study, we used PKC inhibitors, which inhibit predominantly conventional PKC isozymes. Many PKC isozymes, including conventional ones, were identified in the retina and were activated as a result of IR [46]. Since: 1) PKC family is directly involved in the Tlr4-mediated innate immune response [26]; 2) PKC inhibitors reduce retinal damage and inflammation and; 3) release of Hsp70, a known DAMP and ligand of Tlr4 [3,9], was observed after retinal IR, we hypothesized that Hsp70 released as a result of IR activates Tlr4 and initiates a neurotoxic pro-inflammatory response in the presence of PKC activity. To test this hypothesis, we turned to cell cultures. We used Hsp70 containing low-dose endotoxin. However, since endotoxins were still present in Hsp70 preparations, we have taken all precautions to properly interpret the data. We found that the presence of native Hsp70 was critical in Tlr4-dependent innate immune response and PKC inhibitors reduced Hsp70-mediated production of pro-inflammatory factors. Importantly, LPS-activated cells behaved as if PMB inhibits LPS and PKC, while Hsp70-activated cells behaved as if PMB inhibits only PKC and the presence of low endotoxin levels has no role to play. Thus, these data suggest that PKC activity, but not low-dose endotoxin presence, is critical to mediate an Hsp70-dependent pro-inflammatory response.

**Figure 10:**
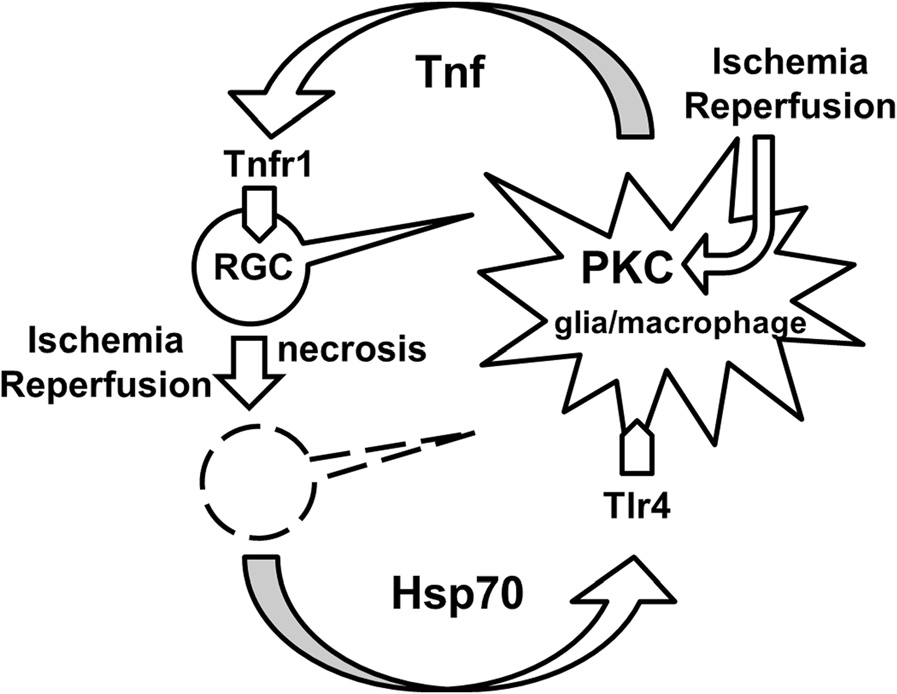
**Proposed mechanism of extracellular Hsp70-mediated neurotoxic pro-inflammatory response in tissue after IR.** The initial ischemic stress followed by reperfusion leads to the cell injury (necrosis) and the release of Hsp70. That same ischemic stress (increase in intracellular Ca^2+^) and reperfusion (increase in ATP (adenosine triphosphate)) mediate PKC activation. These two events are the necessary and sufficient conditions for activation of Tlr4 signaling and production of neurotoxic levels of pro-inflammatory factors such as Tnf. This pro-inflammatory response can trigger further tissue damage leading to increasing levels of extracellular Hsp70. Thus, a cycle may be repeated many times, which can result in significant tissue damage after IR. ATP, Hsp70, heat shock protein 70; IR, ischemic reperfusion; PKC, protein kinase C.

Taken together, we can suggest that PKC activation as a result of ischemia (increase in intracellular Ca^2+^) reperfusion (increase ATP) [46,47] and increase of extracellular Hsp70 as a result of tissue damage promotes the activation of Tlr4 signaling that initiates a neurotoxic pro-inflammatory response (Figure 10). It is known that Myd88 and Trif adaptor proteins modulate the Tlr4 signaling pathway [10,35]. Our data suggests that the Myd88 signaling cascade is probably more important in Hsp70-dependent pro-inflammatory activation of glial cells than the Trif signaling cascade. We also demonstrated that Hsp70 mediates activation of NF-κB transcription factor, which initiates transcription of genes coding proteins such as cytokines, chemokines, and ROS producing enzymes, which can mediate cell death and promote tissue damage [48]. We have found that Hsp70-treated astrocytes are a primary source of chemokines, and thus can facilitate leukocyte infiltration in the ischemic tissue. At the same time, Hsp70-activated microglia may be responsible mainly for ROS production. Both types of glial cells release Tnf and produce the immature form of Il1b in a Tlr4-dependent manner in the presence of Hsp70. Collectively, all these factors can mediate neuronal toxicity, which was observed previously [28,33,49]. Our *in vivo* and *in vitro* data indicate that one way that extracellular Hsp70 can promote neuronal death is by mediating production of cytotoxic levels of Tnf by glial cells. Importantly, in contrast to the data above, intracellular Hsp70 assists protein-folding processes in all cells, and helps to protect neurons from ischemic stress [31,32]. However, the results presented in this study suggest that high levels of extracellular Hsp70 released as a result of ischemic stress in tissue can initiate a strong neurotoxic pro-inflammatory response, which far exceeds the neuroprotective activity of Hsp70.

## Conclusions

In this study we describe for the first time the molecular mechanism of extracellular Hsp70 pro-inflammatory activity in the retina after IR. Our findings indicate that Hsp70 can mediate Tlr4-dependent innate immune response only if PKC activity is present. This data is of great significance because lately there have been doubts regarding the ability of pure DAMPs (extracellular Hsp70 is one of them) to initiate an innate immune response. The mechanism described here can offer insights on the ability of many other DAMPs to mediate innate immune response in ischemic tissue, and may be helpful for the development of new drugs against IR injury.

## Abbreviations

CNS: central nervous system; DAMPs: damage-associated molecular patterns; GCL: ganglion cell layer; Hsp70: heat shock protein 70; Il1b: interleukin-1 β; IOP: intraocular pressure; IP: intraperitoneally; IR: ischemic-reperfusion; Myd88: myeloid differentiation primary response 88; NeuN: neuronal nuclei; NF-κB: nuclear factor kappa B; PKC: protein kinase C; PMB: polymyxin B; RGC: retinal ganglion cell; Tlr:: toll-like receptor; Tnf: tumor necrosis factor; Trif: TIR-domain-containing adapter-inducing interferon-β; VH: vitreous humor; WT: wild type.

## Competing interests

The authors declare they have no competing interests.

## Authors’ contributions

DI and GD conceived the study and participated in its design and coordination. DI, GD, ARCS and AMS analyzed the data, and drafted the manuscript. GD was responsible for induction of IR, retina isolations, immunohistochemistry. XD and ARCS carried out immunohistochemistry, and RGC quantification. DI and AMS carried out quantitative RT-PCR. All authors read and approved the final manuscript.
